# Platelet-Derived Exosomes and Atherothrombosis

**DOI:** 10.3389/fcvm.2022.886132

**Published:** 2022-04-15

**Authors:** Kangkang Wei, Hongbo Huang, Min Liu, Dazhuo Shi, Xiaojuan Ma

**Affiliations:** ^1^National Clinical Research Center for Chinese Medicine Cardiology, Xiyuan Hospital, China Academy of Chinese Medical Sciences, Beijing, China; ^2^Peking University Traditional Chinese Medicine Clinical Medical School (Xiyuan), Beijing, China; ^3^Department of Integrated Chinese and Western Medicine, Peking University Health Science Center, Beijing, China; ^4^Beijing University of Chinese Medicine, Beijing, China

**Keywords:** platelet, exosomes, intercellular communication, atherothrombosis, plaque, thrombus

## Abstract

Platelet-derived exosomes (PLT-Exos) are the main subtype of extracellular vesicles secreted by platelets, which carry proteins, nucleotides, lipids, and other substances to acceptor cells, playing an important role in intercellular communication. PLT-Exos increase with platelet activation and are involved in the process of atherothrombosis by delivering cargo to acceptor cells. Atherosclerotic plaque rupture, causing thrombosis and arterial occlusion, is the basic pathological change leading to cardiovascular events. PLT-Exos from different donors have different functions. PLT-Exos secreted by healthy volunteer or mice can inhibit platelet activation and inflammation of endothelial cells, thus exerting an antithrombotic effect, while PLT-Exos derived from some patients induce endothelial apoptosis and an inflammatory response to promote atherothrombosis. Furthermore, increased PLT-Exos reflect platelet activation and their cargoes also are derived from platelets; therefore, PLT-Exos can also be used as a biomarkers for the diagnosis and prognosis of cardiovascular disease. This article reviews the characteristics of PLT-Exos and discusses their role in cell-to-cell communication and atherothrombosis.

## Introduction

The fundamental mechanism of atherothrombosis comprises plaque disruption and subsequent thrombus formation. Atherothrombotic events, such as myocardial infarction are major causes of cardiovascular death (1). Atherosclerosis starts with endothelial dysfunction, followed by neointima formation, lipid accumulation, foam cell formation, and plaque rupture (2–5). After plaque rupture, prothrombotic substances are exposed to the blood, followed by platelets and coagulation cascade activation, resulting in thrombosis (6–8). In this process, platelets are activated by inflammatory cells, collagen, von Willebrand factor (VWF), tissue factors, and thrombin (9, 10). Platelet activation causes more platelet-derived exosomes (PLT-Exos) to be secreted, which play important roles in atherothrombosis.

Platelet-derived exosomes are a type of extracellular vesicles (EVs), comprising a tiny vesicles with a lipid bilayer released by platelets. More than 75% of EVs, including exosomes, in the blood are derived from platelets (11). Exosomes (30–150 nm in diameter) are derived from the nucleosome and are released by the fusion of multivesicular bodies (MVBs) with the plasma membrane. After release from the donor, exosomes can transport various substances, including mRNAs, microRNAs (miRNAs), proteins, lipids, molecules, ceramide, and phosphatidylserine, to acceptor cells. On the one hand, exosomes play a role in cell-to-cell communication (12–14)in many pathological processes, such as cardiovascular disease (15), body immunity (16), nerve repair (17), aging (18), and cancer (19, 20). On the other hand, exosomal cargoes reflect the status of the parent cells and are important disease diagnostic markers.

Platelets, originating from megakaryocytes in the bone marrow, are an important part of the blood, and are involved in various pathological processes, such as hemostasis, thrombosis, and the immune response (21). During atherothrombosis, platelet activation is accompanied by massive release of PLT-Exos, which in intercellular communication by transporting cargoes such as microRNAs and proteins. Studies have shown that the functions of PLT-Exos from different donors vary. Exosomes secreted by healthy volunteer or mice can inhibit platelet aggregation and endothelial cell inflammation, while PLT-Exos derived from some patients promote endothelial cell apoptosis and the neutrophil-mediated inflammatory response. Based on their important regulatory role, PLT-Exos are expected to be a new method or target for the prevention and treatment of atherothrombosis (22–24). This article reviews the mechanisms involved in the regulation of atherothrombosis by PLT-Exos.

## Characteristics of Platelet-Derived Exosomes

The surface of exosomes comprises different types of surface proteins, such as quad transmembrane proteins, integrins, and immunomodulatory proteins (25). Exosomes can be recognized by most cells and transport proteins, RNAs, cytokines, lipids and other cargoes to acceptor cells to exert multiple regulatory roles (26). Therefore, PLT-Exos usually carry intra platelet substances for information transmission to regulate their target cells. In addition, platelets contain three main types of granules: α granules, dense granules, and lysosomes. Among them, α granules are the most abundant organelles, containing immunoinflammatory regulators, cell adhesive molecules (e.g., fibrinogen, VWF, and multimerin 1), and coagulation factors (e.g., factor V, IX, and XIII) (21). Platelet dense granules are released into the extracellular space directly or through the open canalicular system (OCS) (27) after platelet activation (28). Similar to α molecules, PLT-Exos are also rich in proinflammatory and immunochemokines, such as C-X-C motif chemokine ligand (CXCL)3, C-C motif chemokine ligand 5 (CCL5), CXCL7, platelet factor 4 (PF4), glycoprotein Ib platelet subunit alpha (GP1B), complements C3 and C5, and the platelet activation marker selectin P (CD62p) (29). In addition, platelets can also selectively release RNA into exosomes, prompting exosomes to exert specific functions after entering the acceptor cells (30).

The functions of PLT-Exos are also closely related to causes of platelet activation. Platelet activation caused by different factors results in the release different PLT-Exos in terms of number, size, and, content (31, 32). Platelets produce vesicles with different properties under mechanical forces, such as high shear forces, or in the presence of biochemical reagents, such as thrombin (33, 34). For example, activation of platelets by ADP, thrombin, or collagen results in noticeable differences in terms of the proteins in exosomes (35). Therefore, platelets activated in different diseases can release specific exosomes that can be used for both disease diagnosis and prognostic evaluation, and are directly involved in disease progression.

## Intercellular Communication of Platelet-Derived Exosomes

### The Biogenesis of Platelet-Derived Exosomes

**FIGURE 1:**
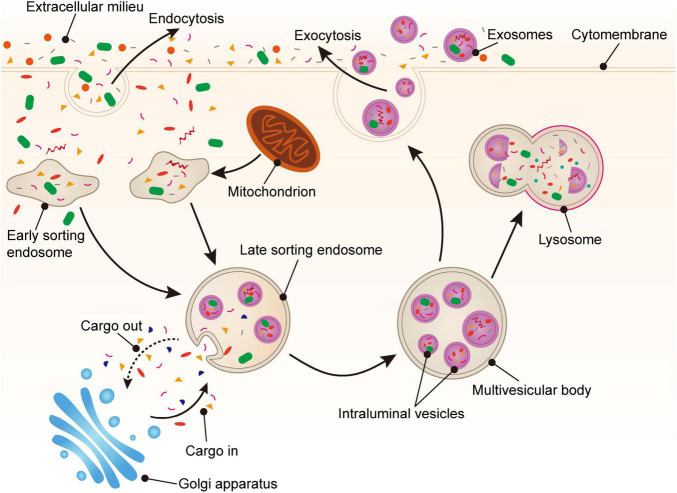
Generation of platelet-derived exosomes. Extracellular proteins, lipids, and metabolites enter the cell through endocytosis. At the luminal side of the cell, the plasma membrane bulge from the outside to the inside to form early sorting endosomes (ESEs). Next, ESEs can fuse with other nucleosomes and transport substances through the Golgi apparatus, gradually forming late sorting endosomes (LSEs). Then, LSEs form intraluminal vesicles (ILVs) through plasma membrane invagination. Finally, the cargoes are further modified *via* the endosomal sorting complex required for transport (ESCRT) pathway or non-ESCRT pathway to eventually form multivesicular bodies (MVBs). MVBs fuse with the plasma membrane and secrete exosomes into the extracellular space *via* exocytosis.

The biogenesis of PLT-Exos is complex. Firstly, various proteins, such as exosomal membrane proteins, lipid-anchored outer membrane proteins, and peripheral surface proteins (36), can enter cells *via* endocytosis of the plasma membrane and form early sorting endosomes. Secondly, early sorting endosomes can fuse with nucleosomes containing other intracellular substances to transform them into late sorting endosomes, which can then transport cargoes with the assistance of the endoplasmic reticulum and Golgi apparatus (12, 36, 37). During the process of late sorting endosome formation, some proteins and lipids are packaged to form intraluminal vesicles (ILVs). Next, ILVs germinate inward (38) to form multivesicular bodies (MVBs). Some MVBs combined with lysosomes or autophagosomes are decomposed, and other MVBs fuse with the plasma membrane under the action of linker proteins to undergo exocytosis. Furthermore, there are many small vesicles in the lumen of MVBs. The MVB lumen contents, including the intact vesicles, are released into the extracellular space. These small vesicles carrying various substances are called exosomes, which reach acceptor cells *via* body fluids, recognize receptors, and enter cells (39, 40) (Figure 1).

The biogenesis of exosomes is regulated by a variety of mechanisms, and both the endosomal sorting complex required for transport (ESCRT) pathway and non-ESCRT pathway play important roles in the formation of MVBs from ILVs. ESCRT is mainly composed of four different protein complexes (ESCRT-0, −I, −II, −III) on the MVB membrane, which encapsulates cargoes through microdomains to form small membrane vesicles and further form ILVs (37, 39). ESCRT-0, which gathers in the limiting membrane of MVBs, can recognize ubiquitinated proteins (cargo) and associate with clathrin. Subsequently, ESCRT-I and ESCRT-II together form stable hetero oligomers with ESCRT-0, aggregating the ubiquitinated cargo in the endosomal membrane. The total complex then recruits and combines with ESCRT-III (23). ESCRT-III promotes the production of complexes (41), ultimately enclosing ILVs into endosomes through budding and dividing. Meanwhile, there is also an ESCRT-independent mechanism for the release of exosomes that still form ILVs, even when all four key subunits of the ESCRT-complex are silenced (42). For example, the inhibition of neutral sphingomyelinase can reduce the release of MVBs and promote the release of exosomes *via* the ESCRT independent pathway (43). In addition, Baietti et al. (44) reported that the syndecan–syntenin–ALG-2-interacting protein X (ALIX) axis is an important regulator of membrane trafficking and heparan sulfate-assisted signaling, which can regulate the occurrence of exosomes.

### Fate of Platelet-Derived Exosomes in Recipient Cells

**FIGURE 2:**
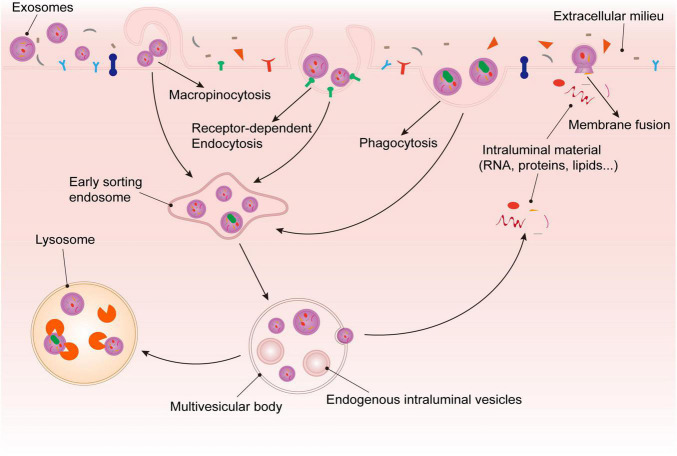
Fate of platelet-derived exosomes in recipient cells. After being recognized by recipient cell surface receptors, exosomes enter cells through *via* phagocytosis, macropinocytosis, membrane fusion, or receptor dependent endocytosis. On the one hand, exosomes that enter *via* membrane fusion release their cargoes into the target cells directly. On the other hand, the exosomes that enter the cell *via* the other methods are internalized to form ESEs, and then combine with ILVs to form MVBs. Some of the exosomal substances are released from MVBs, and the rest would be degraded by lysosomes.

Platelet-Derived Exosomes play a role in cell-to-cell communication by entering acceptor cells to release a variety of substances carried from the mother cells. Exosomes enter recipient cells in four main ways, including receptor-dependent endocytosis, phagocytosis, macropinocytosis, and membrane fusion (45, 46). In the process of entering the cell, exosomes bind to cell surface receptors and move in a slow drifting mode on the plasma membrane, and then enter the cell through endocytosis. Then, exosomes diffuse in the local microenvironment of the cytoplasm in a confined mode or move along the cytoskeleton in rapid directed mode (47, 48). Some exosomes undergoing plasma membrane fusion to release their cargo directly into the acceptor cells, and others enter the cells to form MVBs together with ILVs. One part of MVBs are dissolved by lysosomes, and the other part release their exosomal cargoes into the recipient cells (37) (Figure 2).

## Platelet-Derived Exosomes Regulate Atherothrombosis

### Platelets in Atherothrombosis

Platelets are involved in plaque formation and thrombosis, through platelet adhesion, activation, and aggregation. Platelet adhesion mainly occurs after endothelial cell injury. Platelet glycoprotein GPIa (GPIa) and GPIIa on the platelet membrane bind to collagen through VWF, so that platelets adhere to the injury site (34, 49) and become an important component of the plaques. Platelet activation is reflected in three aspects. First, after platelet adhesion, collagen binds to VWF, triggering calcium-mediated intraplatelet signals, after which thromboxane A2 and adenosine diphosphate bind to other soluble agonists (such as α-thrombin and epinephrine) to promote platelet activation. Second, inflammatory cells, such as leukocytes (50), neutrophils (51), B cells, and T cells also activate platelets (10). Third, after plaque rupture, with activation of the coagulation cascade, thrombin binds to the G protein-linked protease-activated receptor of platelets to activate platelets. After platelet activation, α-and δ-granules are released into the blood, and the adhesive glycoproteins and hemostatic molecules carried by them promote platelet aggregation (34). Platelet aggregation results from platelet activation leading to enhanced binding of platelet surface GPIIb/IIIa receptors to other adhesion proteins, particularly fibrinogen (FG), which exacerbates thrombin-mediated conversion of fibrinogen to fibrin, thereby promoting thrombosis (6).

Platelets are fundamental in atherothrombosis. Many exosomes are secreted after platelet activation to participate in this pathological process. Proteomics showed that integrin subunit alpha 2b (ITGA2B) and integrin subunit beat 3 (ITGB3) levels were enhanced in PLT-Exos from patients with burns (52). ITGA2B binds to FG to promote platelet activation and blood coagulation, and ITGB3 binding to VWF exerts a rapid hemostatic effect. A study illustrated that PLT-Exos are both a marker of disease status and also contain potentially pathogenic proteins. Srikanthan et al. found that PLT-Exos could reduce platelet activity and adhesion to collagen, reduce CD36 expression, and inhibit platelet aggregation in an FeCl3-induced carotid artery thrombosis model in mice (53). Therefore, potentially, PLT-Exos can be both pathogenic, because of the inclusion of procoagulant proteins, and resistant to platelet activation and aggregation, which might be related to the source of PLT-Exos and their specific cargo proteins.

### Endothelial Cells in Atherothrombosis

The role of endothelial cells in atherothrombosis is divided into two aspects. On the one hand, as the initial factors of plaque formation, endothelial injury and barrier dysfunction, are the basis of pathological changes, such as platelet adhesion, lipid deposition, and foam cell and inflammatory cell aggregation. On the other hand, the healthy endothelium expresses mediators to prevent platelet activation, including nitric oxide (NO), prostacyclin (PGI2), and ectonucleoside triphosphate diphosphohydrolase-1 (E-NTPDase1), and those that inhibit coagulation, such as thrombomodulin, the heparin−antithrombin III system, and tissue factor pathway inhibition (34, 54). Besides, endothelium-derived prostacyclin and platelet-derived thromboxane A2 are considered to be mutually antagonistic components of the dynamic thrombotic balance at the vessel-blood interface, which might regulate atherothrombosis (54, 55). Endothelial cell dysfunction is mainly caused to the activation of endothelial cells, especially type II activation with increased expression of interleukin 1 (IL-1), tumor necrosis factor alpha (TNF-α, and vascular cell adhesion molecule 1 (VCAM-1), which leads to chronic inflammation of the endothelium and accelerates atherothrombosis (56).

Endothelial injury promotes the activation of platelets and secretion of PLT-Exos. PLT-Exos can regulate endothelial cell function by transporting miRNAs. In 2013, Gldlöf et al. found that miR-320b released from activated platelets into endothelial cells inhibited intercellular adhesion molecule 1 (ICAM-1) expression in patients with myocardial infarction; however, the study did not determine whether it entered cells through exosomes (57). Yan et al. demonstrated that thrombin-activated platelets can inhibit ICAM-1 expression in endothelial cells through transporting miRNA-223 in exosomes, and found that miR-223 might inhibit endothelial inflammation by regulating nuclear factor kappa B (NF-κB) and mitogen-activated protein kinase (MAPK) pathways (58). To further explore the role of PLT-Exos in endothelial injury, Wang et al. demonstrated that PLT-Exos inhibited the expression of the target gene ADAM10 (encoding A disintegrin and metalloproteinase domain 10), regulated the NF-κB pathway, downregulated IL-1β, IL-6, TNF-α, triglycerides, and total cholesterol, and inhibited endothelial cell inflammation and lipid deposition by delivering miR-25-3p into endothelial cells (59). Therefore, PLT-Exos can protect endothelial cells *via* miRNA regulation.

Platelet-Derived Exosomes can also synergistically regulate endothelial cells through multiple pathways. For example, PLT-Exos can not only enhance ITGA2B and ITGB3 protein levels (52), but also can activate the Yes1 associated transcriptional regulator (YAP) protein (60). These proteins can all activate the phosphatidylinositol-4,5-bisphosphate 3-kinase (PI3K)/protein kinase B (AKT) signaling pathway, which weakens the inflammation of endothelial cells by inducing autophagy. Moreover, in the same disease, PLT-Exos can also act through multiple pathways to complete complex regulatory mechanisms. In patients with sepsis, the Janiszewski et al. (61) found that PLT-Exos can produce reactive oxygen and induce endothelial cell apoptosis through nicotinamide adenine dinucleotide phosphate (NADPH). Subsequently, their team further found that exosomes secreted by platelets exposed to NO and bacteria induced caspase-3 activation and apoptosis in endothelial cells by producing superoxide, NO, and peroxynitrite, which resulted in endothelial dysfunction (62). That study also revealed that PLT-Exos can induce endothelial dysfunction in addition to inhibiting endothelial cell inflammation, probably because the PLT-Exos were derived from patients. Similarly, in diabetic retinopathy, PLT-Exo secretion is significantly increased and CXCL10 is upregulated, which activate the toll like receptor 4 (TLR4) signaling pathway and induce retinal endothelial injury (63). In brief, PLT-Exos can protect endothelial cells by inhibiting endothelial cell inflammation, but can also lead to vascular dysfunction by inducing endothelial cell apoptosis, mainly depending on the source of PLT-Exos and their content.

### Inflammatory Cells in Atherothrombosis

In atherothrombosis, inflammatory cells are both major participants in plaque formation and can promote thrombosis by activating platelets and the coagulation cascade. On the one hand, plaque formation is a chronic inflammatory process (64), and macrophages and T cells produce a large number of mediators, including proinflammatory cytokines, co-stimulators of immune activation, eicosenoids, reactive oxygen species, and nitrogen species (6, 9). Furthermore, inflammation causes platelet activation and promotes plaque progression. Activated platelets not only secrete PF4, regulated upon activation, normally t-expressed, and presumably secreted (RANTES), macrophage inflammatory protein 1-alpha (MIP-1α), and epithelial-derived neutrophil-activating protein 78 (ENA-78) (65), which promote monocyte aggregation, but also expresses CD40L to regulate the formation of platelet-leukocyte complexes and recruit regulatory T cells (66). In addition, platelet-secreted alpha granules store abundant chemokines, such as CXCL4, which can promote the recruitment and activation of endothelial cells and leukocytes (67). On the other hand, thrombus formation is promoted by inflammatory cells. Leukocytes mediate thrombin activation through the production of tissue factor (TF) and granzyme (68–70), and promote thrombus formation through damage-associated molecular patterns (DAMPs) that promote coagulation system activation (65, 71, 72), platelet activation, and aggregation (73–75).

Inflammatory cells can activate platelets to secrete exosomes and promote thrombosis. A previous study focused on the interaction between neutrophils and PLT-Exos. On the one hand, neutrophils promote thrombosis through procoagulant factors and soluble mediators that induce platelet activation and aggregation in neutrophil EVs. On the other hand, neutrophils promote thrombosis through neutrophil extracellular traps (NETs) that create a scaffold for platelets and other blood cells to attach to Blanch-Ruiz et al. (51). Kuravi et al. found that PLT-Exos can promote neutrophil adhesion to endothelial cells and enhance inflammation through CD62P and CXC-chemokines, which resembles the actions of platelet-derived microvesicles (76). Moreover, excessive activation of immune thrombi during septic shock cause thrombotic inflammation, and PLT-Exos activate the AKT/mechanistic target of rapamycin (mTOR) autophagy pathway to promote the formation of NETs through high-mobility group protein 1 (HMGB1) and/or miR-15b-5p and miR-378a-3p (77). In summary, PLT-Exos promote neutrophil-mediated thrombosis; however, the limited number of previous studies has resulted in a lack direct evidence for a role of PLT-Exos in atherothrombosis.

## Application of Platelet-Derived Exosomes

### Potential in the Treatment

The role of PLT-Exos in atherothrombosis is expected to lead to their application in the treatment of coronary heart disease. Previous studies have demonstrated the role of exosomes in coronary heart disease (78, 79) and found that exosomes can be detected in atherosclerotic plaques (80). Unlike other exosomes, PLT-Exos are secreted in large amounts during platelet activation and can regulate thrombosis through multiple pathways, involving platelets, endothelial cells, and inflammation, which have wide potential for therapeutic intervention (Table 1). PLT-Exos reduced endothelial cell inflammation in Apoe -/- mice (59), inhibited the entry of oxidized low-density lipoprotein and cholesterol into macrophages, restrained foam cell formation (53), and then slowed the process of atherosclerosis. In acute thrombosis, in addition to directly inhibiting platelet activation and adhesion (53), PLT-Exos could transfer into smooth muscle cells and reduce the expression of platelet-derived growth factor receptor-beta (PDGFRβ) to inhibit smooth muscle cell proliferation and regulate vascular smooth muscle cell injury and repair (81). The above studies provide an experimental basis for the application of PLT-Exos in disease treatment.

**TABLE 1:** The role of PLT-Exo in atherothrombosis.

Article(references)	Research target	Functional changes
Qin et al. (52)	Coagulation	Coagulation in burn patients (+)
Srikanthan et al. (53)	Thrombosis	Platelet aggregation (−) CD36 in platelet (−) Occlusive thrombosis (−)
Li et al. (58)	Thrombosis-inflammation response	ICAM-1 (−) NF-κB pathways (−) MAPK pathways (−)
Yao et al. (59)	Endothelial cell inflammation	IL-1β, IL-6, and TNF-α (−) Atherosclerosis (−)
Janiszewski et al. (61)	Endothelial cell apoptosis	NADPH in sepsis (+) Apoptosis rates in sepsis (+)
Gambim et al. (62)	Endothelial cell apoptosis	Caspase-3 activation in sepsis (+) Apoptosis in sepsis (+)
Zhang et al. (63)	Endothelial injury	CXCL10 in diabetic rats (+) TLR4 pathways in diabetic rats (+)
Kuravi et al. (76)	Neutrophil-endothelial cell interactions	Adhesion (+) Inflammatory responses (+)
Jiao et al. (77)	Neutrophil	HMGB1 in septic shock (+) Akt/mTOR pathway in septic shock (+) NETs in septic shock (+)
Poon et al. (83)	Monocytes	IL-6 and NLRP3 during CPB (−) inflammatory responses during CPB (−)
Tan et al. (81)	Vascular smooth muscle cells	PDGFRβ (−)

*NADPH, nicotinamide adenine dinucleotide phosphate; CPB, Cardiac surgery with cardiopulmonary bypass; NETs, neutrophil extracellular traps; PDGFRβ, growth factor receptor-beta; (+), increase; (−), decrease.*

Exosomes can be used not only for disease prevention and control, but also as carriers to transport cargo. Exosomes have unique advantages in that they are not easily cleared by immunization and are well tolerated after exosome injection (25). For example, intravenous injection of PLT-Exos inhibited atherosclerosis progression in mice (59). Exosomes act as cargo carriers to deliver miRNAs, siRNAs, and drugs to receptor cells and play a role in regulating target cells to treat diseases (82). For instance, enhanced levels of miR-223 in PLT-Exos inhibited the inflammation involving monocytes (83). In addition, to solve the problem of stent restenosis, Guan et al. immobilized PLT-Exos on the stent surface using electrostatic recheck, which could improve endothelial function, inhibit the macrophage pro-inflammation (M1 phenotype), and promote their conversion to the anti-inflammatory (M2) phenotype (84). This demonstrated the application prospects of PLT-Exo in cardiovascular biomaterials. Another study found that aspirin inhibited the increase in the levels of chemokines and high-mobility group box 1 (HMGB1) in PLT Exos, but the total amount of PLT-Exos was not changed. That study indicated that antiplatelet drugs do not inhibit exosome secretion, and we expect to further explore the synergistic effect of drugs and exosomes in therapy in a future study (85). In short, PLT-Exos can be applied to the treatment of diseases from multiple perspectives, such as directly in treatment, as cargo carriers, in combination with biomaterials or other drugs; however, research in this area is still in its infancy.

### Emerging Diagnostic Markers

Exosomes are widespread in most biological fluids (86), such as blood, saliva, and urine, and are secreted by cells in physiological or pathological conditions. Exosomal cargoes and characteristics are closely related to disease status, and increased attention has been paid to their role in tumor diagnosis and evaluation (87–89). Thus, research has identified them as potential biomarkers for the study of cardiovascular diseases (90, 91). Moreover, clinical diagnosis and treatment would be facilitated through the detection of exosomes in biological fluids, which would reduce the need for invasive operations and computed tomography radiation. For example, Tan M et al. found that miR-223, miR-339, and miR-21, which are associated with platelet activation, were significantly elevated in PLT-Exos before arterial thrombosis, and thus might represent new predictive biomarkers (81). There are still relatively few studies about PLT-Exo, mainly because of the difficulty in extracting PLT-Exos and controlling the experimental conditions. Plasma exosomes are mainly derived from platelets and can be used to replace PLT-Exos to a certain extent, bringing convenience to clinical applications.

## Conclusions and Perspective

Atherothrombosis is the pathological basis of acute cardiovascular events, and platelet activation is an important condition for thrombosis (92). Therefore, how exosomes released by activated platelets function in thrombosis has become the focus of research attention. Exosomes carry a variety of information from platelets into acceptor cells and function in intercellular communication, which is expected to lead to new therapeutic approaches. Therefore, we discussed the role played by PLT-Exos in atherothrombosis and their mechanisms. By specifically delivering different miRNAs and proteins, PLT-Exos can inhibit platelet activation and aggregation, and reduce endothelial cell inflammatory injury. However, different sources of PLT-Exos act differently, and PLT-Exos from some patients would promote endothelial apoptosis and neutrophil-mediated inflammatory response. Hence, flexible applications and modifications of PLT-Exos have great potential to prevent and treat atherothrombosis (93).

Platelet-derived exosomes can be obtained from different sources, leading to significant differences in their cargoes and functions. Exosomes secreted by platelets in disease states often contain pathogenic factors that can be used as biomarkers for disease diagnosis, but do not necessarily act on receptor cells. For example, PLT-Exos are rich in proinflammatory factors and chemokines, reflecting the activation of platelets, while PLT-Exos may play an antiphlogistic and antithrombotic role in receptor cells. In addition, because PLT-Exos are rich in a variety of cargoes, they can play different roles by carrying different regulators. In previous studies, PLT-Exos secreted in disease states often showed high levels of pathogenic factors and can enter target cells to promote disease progression, while exosomes obtained from healthy volunteer or mice can inhibit platelet activation and endothelial inflammation. Of course, these results are only a summary of the current studies on atherothrombosis and are not absolute. For instance, in patients undergoing cardiac surgery with cardiopulmonary bypass (CPB), increased miR-223 in PLT-Exos can downregulate the expression of IL6 and NLRP3 (encoding NLR family pyrin domain containing 3) in monocytes to inhibit the inflammation induced by CPB (84). Thus, the functions of PLT-Exos depend mainly on their source and cargoes.

Although the mechanisms of PLT-Exos in multiple pathological processes, such as platelet activation and endothelial inflammation injury, have been reported, there are still many problems that need further study because of the complex mechanisms of atherothrombosis and the variety of PLT-Exo cargoes. First, thrombosis is mainly caused by platelet activation and the coagulation cascade (94); however, the mechanism by which PLT-Exos regulate platelet activation is unclear, and there is also a lack of studies on the role of the coagulation system. Second, previous studies have affirmed the therapeutic effect of PLT-Exos by intervening in endothelial cells using PLT-Exos in healthy volunteers; however, there is a lack of intervention experiments with PLT-Exos in patients, which makes it difficult to explain the regulatory mechanism of PLT-Exos in disease. Third, experimentally, PLT Exos are obtained by activating platelets using different protocols, which caused differences in exosomal cargoes and thus introduced experimental errors. Recent research has focused on the function of exosomes and the role of their mediated miRNAs and proteins, which are still some distance away from clinical application. On the one hand, we should develop specifications for obtaining PLT-Exos, study the functions of PLT-Exos, then perform genomics analysis to validate miRNAs and proteins that play a major role, and finally apply PLT-Exos in clinical treatment. On the other hand, we can modify PLT-Exos and use them as carriers to deliver specific drugs or cytokines into receptor cells to exert their functions (95).

Encouragingly, previous studies demonstrated the key role of PLT-Exos in atherothrombosis and revealed part of the mechanism, laying the foundation for next step of research. As exosome research progresses, we look forward to the future application of PLT-Exos as diagnostic markers and intervention mediators in the clinical treatment of cardiovascular diseases, ultimately bringing benefits to patients.

## Author Contributions

KW, HH, and ML structured the manuscript giving contribute to figures and text editing. DS and XM revisited the article implementing the final manuscript form. All authors contributed to the manuscript production and in the final revision.

## Conflict of Interest

The authors declare that the research was conducted in the absence of any commercial or financial relationships that could be construed as a potential conflict of interest.

## Publisher’s Note

All claims expressed in this article are solely those of the authors and do not necessarily represent those of their affiliated organizations, or those of the publisher, the editors and the reviewers. Any product that may be evaluated in this article, or claim that may be made by its manufacturer, is not guaranteed or endorsed by the publisher.

## References

[1] Roth GA Mensah GA Johnson CO Addolorato G Ammirati E Baddour LM GBD-NHLBI-JACC global burden of cardiovascular diseases writing group. global burden of cardiovascular diseases and risk factors, 1990-2019: update from the GBD 2019 study. *J Am Coll Cardiol.* (2020) 76:2982–3021. 10.1016/j.jacc.2020.11.010 33309175 PMC7755038

[2] Geovanini GR Libby P. Atherosclerosis and inflammation: overview and updates. *Clin Sci.* (2018) 132:1243–52. 10.1042/CS20180306 29930142

[3] Bentzon JF Otsuka F Virmani R Falk E. Mechanisms of plaque formation and rupture. *Circ Res.* (2014) 114:1852–66. 10.1161/CIRCRESAHA.114.302721 24902970

[4] Libby P Ridker PM Hansson GK. Progress and challenges in translating the biology of atherosclerosis. *Nature.* (2011) 473:317–25. 10.1038/nature10146 21593864

[5] Jabbari N Nawaz M Rezaie J. Bystander effects of ionizing radiation: conditioned media from X-ray irradiated MCF-7 cells increases the angiogenic ability of endothelial cells. *Cell Commun Signal.* (2019) 17:165. 10.1186/s12964-019-0474-8 31842899 PMC6912994

[6] Vorchheimer DA Becker R. Platelets in atherothrombosis. *Mayo Clin Proc.* (2006) 8:59–68. 10.4065/81.1.59 16438480

[7] Sato Y Hatakeyama K Yamashita A Marutsuka K Sumiyoshi A Asada Y. Proportion offibrin and platelets differs in thrombi on ruptured and eroded coronary atherosclerotic plaques in humans. *Heart.* (2005) 91:526–30. 10.1136/hrt.2004.034058 15772220 PMC1768846

[8] Yamashita A Sumi T Goto S Hoshiba Y Nishihira K Kawamoto R Detection of von Willebrand factor and tissue factor in platelets-fibrin rich coronary thrombi in acute myocardial infarction. *Am J Cardiol.* (2006) 97:26–8. 10.1016/j.amjcard.2005.07.105 16377278

[9] Zaverio MR. Platelets in atherothrombosis. *Nat Med.* (2002) 8:1227–34. 10.1038/nm1102-1227 12411949

[10] Koupenova M Clancy L Corkrey HA Freedman JE. Circulating platelets as mediators of immunity, inflammation, and thrombosis. *Circ Res.* (2018) 122:337–51. 10.1161/CIRCRESAHA.117.310795 29348254 PMC5777300

[11] Arraud N Linares R Tan S Gounou C Pasquet JM Mornet S Extracellular vesicles from blood plasma: determination of their morphology, size, phenotype and concentration. *J Thromb Haemost.* (2014) 12:614–27. 10.1111/jth.12554 24618123

[12] Mathieu M Martin-Jaular L Lavieu G Théry C. Specificities of secretion and uptake of exosomes and other extracellular vesicles for cell-to-cell communication. *Nat Cell Biol.* (2019) 21:9–17. 10.1038/s41556-018-0250-9 30602770

[13] Rezaie J Aslan C Ahmadi M Zolbanin NM Kashanchi F Jafari R. The versatile role of exosomes in human retroviral infections: from immunopathogenesis to clinical application. *Cell Biosci.* (2021) 11:19. 10.1186/s13578-021-00537-0 33451365 PMC7810184

[14] Hassanpour M Rezabakhsh A Rezaie J Nouri M Rahbarghazi R. Exosomal cargos modulate autophagy in recipient cells *via* different signaling pathways. *Cell Biosci.* (2020) 10:92. 10.1186/s13578-020-00455-7 32765827 PMC7395405

[15] Saheera S Jani VP Witwer KW Kutty S. Extracellular vesicle interplay in cardiovascular pathophysiology. *Am J Physiol Heart Circ Physiol.* (2021) 320:H1749–61. 10.1152/ajpheart.00925.2020 33666501 PMC8163654

[16] Meldolesi J. Extracellular vesicles, news about their role in immune cells: physiology, pathology and diseases. *Clin Exp Immunol.* (2019) 196:318–27. 10.1111/cei.13274 30756386 PMC6514371

[17] Ching RC Wiberg M Kingham PJ. Schwann cell-like differentiated adipose stem cells promote neurite outgrowth *via* secreted exosomes and RNA transfer. *Stem Cell Res Ther.* (2018) 9:266. 10.1186/s13287-018-1017-8 30309388 PMC6182785

[18] Ahmadi M Rezaie J. Ageing and mesenchymal stem cells derived exosomes: molecular insight and challenges. *Cell Biochem Funct.* (2021) 39:60–6. 10.1002/cbf.3602 33164248

[19] Lazar S Goldfinger LE. Platelets and extracellular vesicles and their cross-talk with cancer. *Blood.* (2021) 137:3192–200. 10.1182/blood.2019004119 33940593 PMC8351904

[20] Jafari R Rahbarghazi R Ahmadi M Hassanpour M Rezaie J. Hypoxic exosomes orchestrate tumorigenesis: molecular mechanisms and therapeutic implications. *J Transl Med.* (2020) 18:474. 10.1186/s12967-020-02662-9 33302971 PMC7731629

[21] Xu XR Zhang D Oswald BE Carrim N Wang X Hou Y Platelets are versatile cells: new discoveries in hemostasis, thrombosis, immune responses, tumor metastasis and beyond. *Crit Rev Clin Lab Sci.* (2016) 53:409–30. 10.1080/10408363.2016.1200008 27282765

[22] Femminò S Penna C Margarita S Comità S Brizzi MF Pagliaro P. Extracellular vesicles and cardiovascular system: biomarkers and cardioprotective effectors. *Vascul Pharmacol.* (2020) 135:106790. 10.1016/j.vph.2020.106790 32861822

[23] Wang H Xie Y Salvador AM Zhang Z Chen K Li G Exosomes: multifaceted messengers in atherosclerosis. *Curr Atheroscler Rep.* (2020) 22:57. 10.1007/s11883-020-00871-7 32772195

[24] Boulanger CM Loyer X Rautou PE Amabile N. Extracellular vesicles in coronary artery disease. *Nat Rev Cardiol.* (2017) 14:259–72. 10.1038/nrcardio.2017.7 28150804

[25] Kalluri R LeBleu VS. The biology, function, and biomedical applications of exosomes. *Science.* (2020) 367:eaau6977. 10.1126/science.aau6977 32029601 PMC7717626

[26] Yáñez-Mó M Siljander PR Andreu Z Zavec AB Borràs FE Buzas EI Biological properties of extracellular vesicles and their physiological functions. *J Extracell Vesicles.* (2015) 4:27066. 10.3402/jev.v4.27066 25979354 PMC4433489

[27] Choi W Karim ZA Whiteheart SW. Protein expression in platelets from six species that differ in their open canalicular system. *Platelets.* (2010) 21:167–75. 10.3109/09537101003611385 20196629 PMC3982968

[28] Heijnen H van der Sluijs P. Platelet secretory behaviour: as diverse as the granules or not? *J Thromb Haemost.* (2015) 13:2141–51. 10.1111/jth.13147 26391322

[29] De Paoli SH Tegegn TZ Elhelu OK Strader MB Patel M Diduch LL Dissecting the biochemical architecture and morphological release pathways of the human platelet extracellular vesiculome. *Cell Mol Life Sci.* (2018) 75:3781–801. 10.1007/s00018-018-2771-6 29427073 PMC11105464

[30] Preußer C Hung LH Schneider T Schreiner S Hardt M Moebus A Selective release of circRNAs in platelet-derived extracellular vesicles. *J Extracell Vesicles.* (2018) 7:1424473. 10.1080/20013078.2018.1424473 29359036 PMC5769804

[31] Aatonen MT Ohman T Nyman TA Laitinen S Grönholm M Siljander PR. Isolation and characterization of platelet-derived extracellular vesicles. *J Extracell Vesicles.* (2014) 3:1. 10.3402/jev.v3.24692 25147646 PMC4125723

[32] Leong HS Podor TJ Manocha B Lewis JD. Validation of flow cytometric detection of platelet microparticles and liposomes by atomic force microscopy. *J Thromb Haemost.* (2011) 9:2466–76. 10.1111/j.1538-7836.2011.04528.x 21981726

[33] Reininger AJ Heijnen HF Schumann H Specht HM Schramm W Ruggeri ZM. Mechanism of platelet adhesion to von willebrand factor and microparticle formation under high shear stress. *Blood.* (2006) 107:3537–45. 10.1182/blood-2005-02-0618 16449527 PMC1895770

[34] Aatonen M Grönholm M Siljander PR. Platelet-derived microvesicles: multitalented participants in intercellular communication. *Semin Thromb Hemost.* (2012) 38:102–13. 10.1055/s-0031-1300956 22314608

[35] Milioli M Ibáñez-Vea M Sidoli S Palmisano G Careri M Larsen MR. Quantitative proteomics analysis of platelet-derived microparticles reveals distinct protein signatures when stimulated by different physiological agonists. *J Proteomics.* (2015) 121:56–66. 10.1016/j.jprot.2015.03.013 25835965

[36] Pegtel DM Gould SJ. Exosomes. *Annu Rev Biochem.* (2019) 88:487–514. 10.1146/annurev-biochem-013118-111902 31220978

[37] van Niel G D’Angelo G Raposo G. Shedding light on the cell biology of extracellular vesicles. *Nat Rev Mol Cell Biol.* (2018) 19:213–28. 10.1038/nrm.2017.125 29339798

[38] Huotari J Helenius A. Endosome maturation. *EMBO J.* (2011) 30:3481–500. 10.1038/emboj.2011.286 21878991 PMC3181477

[39] Hurley JH. ESCRTs are everywhere. *EMBO J.* (2015) 34:2398–407. 10.15252/embj.201592484 26311197 PMC4601661

[40] Colombo M Raposo G Théry C. Biogenesis, secretion, and intercellular interactions of exosomes and other extracellular vesicles. *Annu Rev Cell Dev Biol.* (2014) 30:255–89. 10.1146/annurev-cellbio-101512-122326 25288114

[41] Teis D Saksena S Emr SD. Ordered assembly of the ESCRT-III complex on endosomes is required to sequester cargo during MVB formation. *Dev Cell.* (2008) 15:578–89. 10.1016/j.devcel.2008.08.013 18854142

[42] Stuffers S Sem WC Stenmark H Brech A. Multivesicular endosome biogenesis in the absence of ESCRTs. *Traffic.* (2009) 10:925–37. 10.1111/j.1600-0854.2009.00920.x 19490536

[43] Trajkovic K Hsu C Chiantia S Rajendran L Wenzel D Wieland F Ceramide triggers budding of exosome vesicles into multivesicular endosomes. *Science.* (2008) 319:1244–7. 10.1126/science.1153124 18309083

[44] Baietti MF Zhang Z Mortier E Melchior A Degeest G Geeraerts A Syndecan-syntenin-ALIX regulates the biogenesis of exosomes. *Nat Cell Biol.* (2012) 14:677–85. 10.1038/ncb2502 22660413

[45] Mulcahy LA Pink RC Carter DR. Routes and mechanisms of extracellular vesicle uptake. *J Extracell Vesicles.* (2014) 3:1. 10.3402/jev.v3.24641 25143819 PMC4122821

[46] Morrison EE Bailey MA Dear JW. Renal extracellular vesicles: from physiology to clinical application. *J Physiol.* (2016) 594:5735–48. 10.1113/JP272182 27104781 PMC5063943

[47] Svensson KJ Christianson HC Wittrup A Bourseau-Guilmain E Lindqvist E Svensson LM Exosome uptake depends on ERK1/2-heat shock protein 27 signaling and lipid Raft-mediated endocytosis negatively regulated by caveolin-1. *J Biol Chem.* (2013) 288:17713–24. 10.1074/jbc.M112.445403 23653359 PMC3682571

[48] Tian T Zhu YL Hu FH Wang YY Huang NP Xiao ZD. Dynamics of exosome internalization and trafficking. *J Cell Physiol.* (2013) 22:1487–95. 10.1002/jcp.24304 23254476

[49] Savage B Saldivar E Ruggeri ZM. Initiation of platelet adhesion by arrest onto fibrinogen or translocation on von Willebrand factor. *Cell.* (1996) 84:289–97. 10.1016/s0092-8674(00)80983-6 8565074

[50] Swystun LL Liaw PC. The role of leukocytes in thrombosis. *Blood.* (2016) 128:753–62. 10.1182/blood-2016-05-718114 27354721

[51] Blanch-Ruiz MA Ortega-Luna R Martínez-Cuesta MÁ Álvarez Á. The neutrophil secretome as a crucial link between inflammation and thrombosis. *Int J Mol Sci.* (2021) 22:4170. 10.3390/ijms22084170 33920656 PMC8073391

[52] Qin D Yang W Pan Z Zhang Y Li X Lakshmanan S. Differential proteomics analysis of serum exosomein burn patients. *Saudi J Biol Sci.* (2020) 27:2215–20. 10.1016/j.sjbs.2020.06.024 32874118 PMC7451684

[53] Srikanthan S Li W Silverstein RL McIntyre TM. Exosome poly-ubiquitin inhibits platelet activation, downregulates CD36 and inhibits pro-atherothombotic cellular functions. *J Thromb Haemost.* (2014) 12:1906–17. 10.1111/jth.12712 25163645 PMC4229405

[54] Bochenek ML Schäfer K. Role of endothelial cells in acute and chronic thrombosis. *Hamostaseologie.* (2019) 39:128–39. 10.1055/s-0038-1675614 30620989

[55] Etulain J Schattner M. Glycobiology of platelet-endothelial cell interactions. *Glycobiology.* (2014) 24:1252–9. 10.1093/glycob/cwu056 24928621

[56] Gimbrone MAJ García-Cardeña G. Endothelial cell dysfunction and the pathobiology of atherosclerosis. *Circ Res.* (2016) 118:620–36. 10.1161/CIRCRESAHA.115.306301 26892962 PMC4762052

[57] Gidlöf O van der Brug M Ohman J Gilje P Olde B Wahlestedt C Platelets activated during myocardial infarction release functional miRNA, which can be taken up by endothelial cells and regulate ICAM1 expression. *Blood.* (2013) 121:3908–17. 10.1182/blood-2012-10-461798 23493781

[58] Li J Tan M Xiang Q Zhou Z Yan H. Thrombin-activated platelet-derived exosomes regulate endothelial cell expression of ICAM-1 *via* microRNA-223 during the thrombosis-inflammation response. *Thromb Res.* (2017) 154:96–105. 10.1016/j.thromres.2017.04.016 28460288

[59] Yao Y Sun W Sun Q Jing B Liu S Liu X Platelet-derived exosomal MicroRNA-25-3p inhibits coronary vascular endothelial cell inflammation through adam10 *via* the NF-κB signaling pathway in ApoE-/- Mice. *Front Immunol.* (2019) 10:2205. 10.3389/fimmu.2019.02205 31632389 PMC6783608

[60] Zhang W Jiang H Kong Y. Exosomes derived from platelet-rich plasma activate YAP and promote the fibrogenic activity of müller cells *via* the PI3K/Akt pathway. *Exp Eye Res.* (2020) 193:107973. 10.1016/j.exer.2020.107973 32059976

[61] Janiszewski M Do CAO Pedro MA Silva E Knobel E Laurindo FR. Platelet-derived exosomes of septic individuals possess proapoptotic NAD(P)H oxidase activity: a novel vascular redox pathway. *Crit Care Med.* (2004) 32:818–25. 10.1097/01.ccm.0000114829.17746.19 15090968

[62] Gambim MH do CAO Marti L Veríssimo-Filho S Lopes LR Janiszewski M. Platelet-derived exosomes induce endothelial cell apoptosis through peroxynitrite generation: experimental evidence for a novel mechanism of septic vascular dysfunction. *Crit Care.* (2007) 11:R107. 10.1186/cc6133 17894858 PMC2556756

[63] Zhang W Dong X Wang T Kong Y. Exosomes derived from platelet-rich plasma mediate hyperglycemia-induced retinal endothelial injury *via* targeting the TLR4 signaling pathway. *Exp Eye Res.* (2019) 189:107813. 10.1016/j.exer.2019.107813 31560926

[64] Hansson GK. Inflammation, atherosclerosis, and coronary artery disease. *N Engl J Med.* (2005) 352:1685–95. 10.1056/NEJMra043430 15843671

[65] von Brühl ML Stark K Steinhart A Chandraratne S Konrad I Lorenz M Monocytes, neutrophils, and platelets cooperate to initiate and propagate venous thrombosis in mice *in vivo*. *J Exp Med.* (2012) 209:819–35. 10.1084/jem.20112322 22451716 PMC3328366

[66] Lievens D Zernecke A Seijkens T Soehnlein O Beckers L Munnix IC Platelet CD40L mediates thrombotic and inflammatory processes in atherosclerosis. *Blood.* (2010) 116:4317–27. 10.1182/blood-2010-01-261206 20705757 PMC2993630

[67] Gleissner CA. Platelet-derived chemokines in atherogenesis: what’s new? *Curr Vasc Pharmacol.* (2012) 10:563–9. 10.2174/157016112801784521 22338571

[68] Shantsila E Lip GYH. The role of monocytes in thrombotic disorders. Insights from tissue factor, monocyte-platelet aggregates and novel mechanisms. *Thromb Haemost.* (2009) 102:916–24. 10.1160/TH09-01-0023 19888530

[69] Gale AJ Rozenshteyn D. Cathepsin G, a leukocyte protease, activates coagulation factor VIII. *Thromb Haemost.* (2008) 99:44–51. 10.1160/TH07-08-0495 18217133

[70] Plescia J Altieri DC. Activation of Mac-1(CD11b/CD18)-bound factor X by released cathepsin G defines an alternative pathway of leucocyte initiation of coagulation. *Biochem J.* (1996) 319:873–9. 10.1042/bj3190873 8920993 PMC1217869

[71] Fuchs TA Brill A Wagner DD. Neutrophil extracellular trap (NET) impact on deep vein thrombosis. *Arterioscler Thromb Vasc Biol.* (2012) 32:1777–83. 10.1161/ATVBAHA.111.242859 22652600 PMC3495595

[72] Gould TJ Vu TT Swystun LL Dwivedi DJ Mai SH Weitz JI Neutrophil extracellular traps promote thrombin generation through platelet-dependent and platelet-independent mechanisms. *Arterioscler Thromb Vasc Biol.* (2014) 34:1977–84. 10.1161/ATVBAHA.114.304114 25012129

[73] Renesto P Chignard M. Enhancement of cathepsin G-induced platelet activation by leukocyte elastase: consequence for the neutrophil-mediated platelet activation. *Blood.* (1993) 82:139–44. 10.1182/blood.V82.1.139 8324217

[74] LaRosa CA Rohrer MJ Benoit SE Rodino LJ Barnard MR Michelson AD. Human neutrophil cathepsin G is a potent platelet activator. *J Vasc Surg.* (1994) 19:306–19. 10.1016/s0741-5214(94)70106-7 7509416

[75] Yan SL Russell J Granger DN. Platelet activation and platelet-leukocyte aggregation elicited in experimental colitis are mediated by interleukin-6. *Inflamm Bowel Dis.* (2014) 20:353–62. 10.1097/01.MIB.0000440614.83703.84 24390064 PMC3947085

[76] Kuravi SJ Harrison P Rainger GE Nash GB. Ability of platelet-derived extracellular vesicles to promote neutrophil-endothelial cell interactions. *Inflammation.* (2019) 42:290–305. 10.1007/s10753-018-0893-5 30218321 PMC6394582

[77] Jiao Y Li W Wang W Tong X Xia R Fan J Platelet-derived exosomes promote neutrophil extracellular trap formation during septic shock. *Crit Care.* (2020) 24:380. 10.1186/s13054-020-03082-3 32600436 PMC7322900

[78] Gao XF Wang ZM Wang F Gu Y Zhang JJ Exosomes in coronary artery disease. *Int J Biol Sci.* (2019) 15:2461–70. 10.7150/ijbs.36427 31595163 PMC6775305

[79] Mutlu BR Edd JF Toner M. Oscillatory inertial focusing in infinite microchannels. *Proc Natl Acad Sci USA.* (2018) 115:7682–7. 10.1073/pnas.1721420115 29991599 PMC6065022

[80] Perrotta I Aquila S. Exosomes in human atherosclerosis: an ultrastructural analysis study. *Ultrastruct Pathol.* (2016) 40:101–6. 10.3109/01913123.2016.1154912 27031176

[81] Tan M Yan HB Li JN Li WK Fu YY Chen W Thrombin stimulated platelet-derived exosomes inhibit platelet-derived growth factor receptor-beta expression in vascular smooth muscle cells. *Cell Physiol Biochem.* (2016) 38:2348–65. 10.1159/000445588 27198239

[82] Parodi A Molinaro R Sushnitha M Evangelopoulos M Martinez JO Arrighetti N Bio-inspired engineering of cell- and virus-like nanoparticles for drug delivery. *Biomaterials.* (2017) 147:155–68. 10.1016/j.biomaterials.2017.09.020 28946131

[83] Poon KS Palanisamy K Chang SS Sun KT Chen KB Li PC Plasma exosomal miR-223 expression regulates inflammatory responses during cardiac surgery with cardiopulmonary bypass. *Sci Rep.* (2017) 7:10807. 10.1038/s41598-017-09709-w 28883474 PMC5589826

[84] Hou YC Li JA Zhu SJ Cao C Tang JN Zhang JY Tailoring of cardiovascular stent material surface by immobilizing exosomes for better pro-endothelialization function. *Colloids Surf B Biointerfaces.* (2020) 189:110831. 10.1016/j.colsurfb.2020.110831 32058252

[85] Goetzl EJ Goetzl L Karliner JS Tang N Pulliam L. Human plasma platelet-derived exosomes: effects of aspirin. *FASEB J.* (2016) 30:2058–63. 10.1096/fj.201500150R 26873936 PMC4836374

[86] Simpson RJ Lim JW Moritz RL Mathivanan S. Exosomes: proteomic insights and diagnostic potential. *Expert Rev Proteomics.* (2009) 6:267–83. 10.1586/epr.09.17 19489699

[87] Poulet G Massias J Taly V. Liquid biopsy: general concepts. *Acta Cytol.* (2019) 63:449–55. 10.1159/000499337 31091522

[88] Ramalingam N Jeffrey SS. Future of liquid biopsies with growing technological and bioinformatics studies: opportunities and challenges in discovering tumor heterogeneity with single-cell level analysis. *Cancer J.* (2018) 24:104–8. 10.1097/PPO.0000000000000308 29601337 PMC5880298

[89] Mader S Pantel K. Liquid biopsy: current status and future perspectives. *Oncol Res Treat.* (2017) 40:404–8. 10.1159/000478018 28693023

[90] Jansen F Nickenig G Werner N. Extracellular vesicles in cardiovascular disease: potential applications in diagnosis, prognosis, and epidemiology. *Circ Res.* (2017) 120:1649–57. 10.1161/CIRCRESAHA.117.310752 28495995

[91] Loyer X Vion AC Tedgui A Boulanger CM. Microvesicles as cell-cell messengers in cardiovascular diseases. *Circ Res.* (2014) 114:345–53. 10.1161/CIRCRESAHA.113.300858 24436430

[92] Libby P Theroux P. Pathophysiology of coronary artery disease. *Circulation.* (2005) 111:3481–8. 10.1161/CIRCULATIONAHA.105.537878 15983262

[93] Weber C Badimon L Mach F van der Vorst EPC. Therapeutic strategies for atherosclerosis and atherothrombosis: past, present and future. *Thromb Haemost.* (2017) 117:1258–64. 10.1160/TH16-10-0814 28594053

[94] Koenen RR Binder CJ. Platelets and coagulation factors: established and novel roles in atherosclerosis and atherothrombosis. *Atherosclerosis.* (2020) 307:78–9. 10.1016/j.atherosclerosis.2020.07.008 32718764

[95] Johnson J Wu YW Blyth C Lichtfuss G Goubran H Burnouf T. Prospective therapeutic applications of platelet extracellular vesicles. *Trends Biotechnol.* (2021) 39:598–612. 10.1016/j.tibtech.2020.10.004 33160678

